# Utilizing cyberplace managers to prevent and control cybercrimes: a vignette experimental study

**DOI:** 10.1057/s41284-023-00371-8

**Published:** 2023-03-21

**Authors:** Heemeng Ho, John Gilmour, Lorraine Mazerolle, Ryan Ko

**Affiliations:** 1 Cyber Security, School of Information Technology and Electrical Engineering, Faculty of Engineering, Architecture and Information Technology, The University of Queensland, Brisbane, QLD 4072 Australia; 2 School of Social Science, Faculty of Humanities and Social Sciences, The University of Queensland, Brisbane, QLD 4072 Australia; 3 Information Security, ICT Cluster, Singapore Institute of Technology, 172A Ang Mo Kio Avenue 8, Singapore, 567739 Singapore

**Keywords:** Situational Crime Prevention (SCP), Cybercrime, Cyberspace, Vignette, Place manager, Cyberplace manager

## Abstract

Place management is central to Routine Activities Theory and proactive utilization of place managers is one of 25 Situational Crime Prevention techniques. Yet little is known about the effectiveness of using place managers to prevent cybercrimes. This paper uses a vignette experimental survey of 213 cybersecurity professionals to explore their perceptions of cybercrime prevention. We find that organizations that partner with and proactively utilize cybersecurity professionals as place managers are perceived as more effective in controlling cybercrimes than those organizations that do not specifically assign professionals to proactively prevent and control cybercrimes. We conclude that assigned cyberplace managers are more likely to use their cyber skills and knowledge than those who lack the time and space to utilize their cyber expertise.

## Introduction

Eck (1994) first introduced the concept of “place manager” to describe people who monitor and control criminal behavior at specific places. Examples of place managers are bar owners, parking lot attendants or anyone who is either hired or has ownership claims to the place (Douglas and Welsh 2020). Madensen (2007) stresses that place managers can play an important role in crime prevention in addition to their primary role: for example, bar managers serving alcoholic drinks responsibly or parking lot attendants paying attention to vehicle number plates. Utilizing place managers to prevent crimes is now incorporated as one of the 25 Situational Crime Prevention (SCP) techniques (Cornish and Clarke 2003). SCP is a criminological approach to prevent crimes that has been widely adopted by many governments such as in Australia (Morgan et al. 2012) and the United Kingdom (UK Home Office 2016).

The challenges of preventing and controlling cybercrime present potential new thinking about the role and impact of place managers. Many organizations hire cybersecurity professionals to prevent cybercrimes in their workplaces. These cybersecurity professionals are essentially the “cyber” equivalent of place managers because they monitor and protect their organizations’ cyberplaces. These cyberplaces include physical cyber-infrastructures such as computer servers and devices or virtual places such as websites (Madensen and Eck 2012) or virtual reality worlds. The role of cyberplace managers is becoming increasingly important as cybercrime threats and losses for individuals and organizations grow. For example, the Federal Bureau of Investigation’s (FBI) Internet Crime Complaint Center (2021) registered a record number of almost 850,000 complaints and reported potential cybercrime losses of USD 6.9 billion in 2021. Similarly, PwC Global Digital Trust Insights (2022) reported that 66% of their respondents anticipated an increase in cybercrimes. Therefore, we anticipate a growing interest in organizations’ utilizing cyber personnel as place managers to prevent cybercrimes.

This paper explores the crime prevention perceptions and attitudes of 213 cyber personnel using a novel vignette experimental study. In vignette experimental studies, survey respondents are shown brief descriptions of scenarios or persons (i.e., vignettes), then their judgements on these scenarios are collected (Atzmüller and Steiner 2010). Using this experimental approach, our paper is structured into five main sections. The first section introduces the background, motivations and purpose of the paper. We then review the extant research on place management and the activities of cyberplace managers in preventing cybercrimes in their organizations in the second section. We describe the vignette experimental study in the third section. In the fourth section, we present the results of the vignette experimental study. Finally, we summarize the key results, highlight the limitations, and present directions for future research in this area.

## Literature review

### Research development of place managers

A place is defined as a location (physical or virtual) where human activities occur (Madensen and Eck 2012). Virtual or physical places that involve human activities all create potential for crimes to happen. Eck (1994) first broaches the idea of place managers taking on extra responsibilities to discourage crimes at places. Eck (2003) articulates a triplet of discouragement roles using the crime triangle of Routine Activities Theory (Felson 1995): guardians discourage crimes in situations, intimate handlers discourage crimes by motivated offenders, and place managers discourage crimes in crime prone places. Felson (1995) further discusses four levels of place managers in terms of responsibilities: (i) *personal* responsibility, (ii) *assigned* responsibility, (iii) *diffuse* responsibility, and (iv) *general* responsibility. Place managers with *personal* responsibilities are owners of places, such as homeowners, who exercise personal ownership over their places. Place managers with *assigned* responsibilities are people such as employees who are specifically assigned to look after places. For example, a teacher may be assigned responsibilities to ensure the safety of their students when teaching in a classroom. Place managers with *diffuse* responsibilities are other employed people with less precise responsibilities. For example, a teacher walking in a school corridor may take note of strangers loitering on the school premises. Finally, a passer-by or bystander in the street could be a place manager with *general* responsibility whose casual presence could deter broad daylight crimes such as street murders. The original articulation of place managers discouraging crimes in places focused exclusively on physical places like street corners, parks, shopping centers, public housing sites, and bars. The idea of crimes taking place in cyberspace was not a consideration in the 1990s. Yet, by the turn of the century, the FBI’s Internet Crime Center saw cybercrime complaints grow from almost 50,000 in 2001 to nearly 850,000 in 2021 (National White Collar Crime Center and Federal Bureau of Investigation (FBI) 2002; FBI’s Internet Crime Complaint Center 2021), leading to refreshed thinking about place management.

Madensen and Eck (2012) highlight that existing terminologies to define places are now insufficient, particularly in the era of cybercrimes. They propose three new terms to describe places more precisely: (i) *proprietary*, (ii) *proximal,* and (iii) *pooled*. A *proprietary* place is essentially a place that has distinct owners. This is important because owners have legal authority over the place that they own. Proprietary places can be physical or virtual, for example, a physical apartment owned by a person or a virtual webpage owned by an organization. *Proximal* places are small groups of proprietary places that are located relatively close to one another. In these places, there can be more than one owner. However, no owner has full control over the proximal places, for example, a street block (with no specific owner) containing a row of apartments (with multiple owners) or a website with personal blogs by different individuals. *Pooled* places are arbitrary places with higher levels of proprietary place aggregation. These larger groups of proprietary places generally share certain common characteristics such as a neighborhood or, in the case of cyber aggregations, a data center housing multiple types of websites.

The concept of place managers has also changed over time in the context of SCP. Cornish and Clarke (2003), for example, add “utilizing place managers” as one of the 25 SCP techniques in place of “surveillance by employees” (Clarke and Homel 1997). Douglas and Welsh (2022) highlight that earlier research work in SCP focused on proprietary places such as apartment building managers as place managers (Clarke and Bichler-Robertson 1998). Later, research on place managers extended to bar staff taking on place management responsibility (see, for example, Graham et al. 2004; Madensen and Eck 2008) and using landlords as place managers to control drug and disorder problems for proximal places (Mazerolle et al. 1998). Collaboration between police and place managers in entertainment districts and regulations to foster responsible operators of nuisance motels are other examples of research explicating the role of place managers in physical places (Bichler et al. 2013). Researchers such as Clarke and Bichler-Robertson (1998) and Welsh et al. (2010) highlight that there is little criminological research on the effectiveness of place managers in preventing crimes. In their systematic review of the effects of place managers on crime in public and private spaces, Douglas and Welsh (2022) note that there is favorable evidence showing that using place managers is a promising, albeit underutilized, SCP technique. Yet the use of place managers in preventing and controlling cybercrimes remains absent from the evaluation literature.

The effectiveness of SCP in combating traditional crimes, ranging from robberies to vandalism, is well documented (Hodgkinson and Farrell 2018; Brewer et al. 2019). Additionally, SCP is widely used to combat non-traditional crimes such as wildlife crime (Pires and Clarke 2011; Kurland et al. 2017; Moreto and Gau 2017; Burton et al. 2020), terrorism (Clarke and Newman 2009; Mandala and Freilich 2018; Freilich et al. 2019), and maritime piracy (Shane et al. 2015, 2018). Given the successful implementation of SCP by many governments and its wide adoption in dealing with a variety of traditional and non-traditional crimes, it is unsurprising that there is growing interests in using SCP to prevent cybercrimes (Willison 2000; Beebe and Rao 2005; Willison and Siponen 2009; Hinduja and Kooi 2013; Padayachee 2015; Ho et al. 2022).

Brewer et al. (2019) argue that it is unclear whether SCP interventions can prevent cybercrime effectively. They note that the effectiveness of cybersecurity crime control measures in SCP (hereafter, referred to as “cyber controls”) tend to focus on three limited areas: antivirus, warnings, and formal surveillance software and tools. Ho et al. (2022) conducted a systematic search of studies to identify the SCP techniques used to prevent and control cybercrimes. They note several potential research issues that may have hampered research of SCP in cybercrimes (Ho et al. 2022). Ho et al. (2022) conclude that the human and technical nature of cybercrimes requires an in-depth understanding of both criminology and cybersecurity to apply SCP techniques to prevent and control cybercrimes. The typical cybersecurity professional may be familiar with cybersecurity controls but is unlikely to be familiar with SCP. The reverse is true for many criminologists. This could be one reason why it is difficult for researchers who are not well versed in both SCP and cybersecurity to model and determine suitable cybersecurity controls that can be used in SCP interventions. Additionally, there are calls from researchers (Brewer et al. 2019; Maimon and Louderback 2019; Ho et al. 2022) for more empirical evidence to better understand the effectiveness of SCP in cybercrime prevention. This echoes the earlier calls for more evaluative research into the role of place managers (Welsh et al. 2010; Douglas and Welsh 2020).

With some notable exceptions, exploring the way organizations create and utilize place managers in cyberspace is not well established in the extant literature. One exception is Reyns (2010), who discusses the use of SCP techniques and the role of cyberplace managers in combating cyberstalking: he starts by examining the nature of cyberstalking, victimization, cyberspace, and place management and subsequently proposes preventive measures to combat cyberstalking based on the SCP measures for individuals and place managers. Reyns (2010) argues that place management has a clear equivalent in cyberspace and SCP techniques such as using place managers are applicable to cybercrime prevention. Similarly, Maimon et al. (2022) look further into the role of place managers and note that place managers increase the likelihood of people engaging in self-protective behaviors when using public Wi-Fi networks. Yet the effectiveness of place managers in preventing cybercrimes remains uncertain. This current lack of knowledge around the effectiveness of using place managers in preventing cybercrimes is our primary motivation for conducting this vignette study.

### Cybersecurity activities by cyberplace managers

Organizations are increasingly hiring cybersecurity professionals as assigned cyberplace managers to combat cybercrimes. In the United States, for example, the employment of cybersecurity professionals as cyberplace managers is projected to grow 33% from 2020 to 2030; much faster growth than the average rate for all other occupations (US Bureau of Labor Statistics n.d.). This shows the growing significance of using cybersecurity professionals as assigned cyberplace managers to protect an organization’s assets and prevent cybercrimes. These cyberplace managers typically implement a multitude of cybersecurity controls from popular cybersecurity standards and frameworks such as the ISO/IEC 27002:2022 (ISO/IEC 2022). The number of cybersecurity controls that can be put in place is numerous. ISO/IEC 27002:2022 alone lists a total of 93 controls over four control types. The complexities in cybersecurity and cybercrime prevention in organizations can lead to these cyberplace managers having a diverse set of actions and roles. As a result, a lone assigned cyberplace manager, especially one who oversees many or large cyberplaces, may often have limited time, energy, and resources to implement the myriad of controls needed. As such, organizations may hire more than one assigned cyberplace manager to work in a larger team. Within a team of assigned cyberplace managers, some of them could specialize in different areas of cybersecurity, such as penetration testing, incident response, or digital forensics. Therefore, cyberplace managers can have a variety of work roles and job titles such as Information Security Analyst, Cyber Defense Assessor and Cyber Defense Incident Responder (Petersen et al. 2020; US Bureau of Labor Statistics n.d.).

The work of cybersecurity professionals generally revolves around five key cybersecurity functions (National Institute of Standards and Technology 2018; ISO/IEC 2022): (i) *identify*, (ii) *protect*, (iii) *detect*, (iv) *respond,* and (v) *recover*. *Identify* refers to identifying possible cybersecurity risks to assets such as data and cyber-infrastructures. *Protect* usually means protecting assets and data using appropriate cybersecurity controls. *Detect* is a process of detecting cybersecurity threats. *Respond* refers to reacting to potential cybersecurity threats. *Recover* usually means recovering from cybersecurity incidents, such as a ransomware attack, minimizing losses and restoring various IT functionalities. These five key cybersecurity functions can lead to a variety of cybersecurity actions. For example, in ISO/IEC 27002:2022 the *Identify* cybersecurity function could result in cybersecurity controls such as implementing “Threat Intelligence” (ISO/IEC 27002:2022 Control #5.7), where information relating to information security threats is proactively collected, identified and analyzed so that the organization can take appropriate actions to minimize cybersecurity threats. In another example, the *Respond* cybersecurity function can include controls such as coming into “Contact with Special Interest Groups” (ISO/IEC 27002:2022 Control #5.6), where organizations work with other cybersecurity special interest groups or forums such as the national-level cyber-emergency response agency or Computer Emergency Response Teams (CERTs) that manage cybersecurity incidents.

Organizations may also have limited knowledge, skills, and expertise, as well as finite financial and personnel resources and time restraints, to implement a myriad of cybersecurity controls. For example, as mentioned earlier, there are 93 controls listed in ISO/IEC 27002:2022, while the MITRE ATT&CK matrix (Strom et al. 2018) for the enterprise lists 11 tactics and 291 techniques used by cyber-attackers. As such, organizations may choose to seek assistance from other cybersecurity organizations such as CERT experts, who are known for their skills and expertise in cybersecurity incident management (AusCERT n.d.). Furthermore, popular cybersecurity industry standards such as ISO/IEC 27002:2022 focus more on all-purpose cybersecurity protection rather than on preventing specific cybercrimes (Ho et al. 2022). However, it may be more effective for organizations to channel their limited energy and resources into implementing cybersecurity controls in a more targeted manner to prevent specific cybercrimes, especially those that carry higher risks. For example, the European Union Agency for Cybersecurity (ENISA) Threat Landscape 2021 report assessed ransomware as the prime threat in their reporting period (ENISA 2021). The controls proposed in the industry cybersecurity standards are viewed as anecdotal because they are based on cybersecurity professionals’ working experience rather than empirical evidence to prove their effectiveness (Siponen and Willison 2009). Our paper aims to answer research calls on the lack of empirical evidence on the effectiveness of cyberplace managers (Siponen and Willison 2009; Welsh et al. 2010; Brewer et al. 2019; Maimon and Louderback 2019; Douglas and Welsh 2020; Ho et al. 2022) through a vignette experimental study.

## Methods

### Hypotheses

This paper employs a vignette experimental study to assess the perception of the effectiveness of actions taken by cyberplace managers in controlling cybercrimes, such as ransomware, in their organizations. We examine the perception of effectiveness of two of the cybersecurity actions undertaken by cyberplace managers: (i) utilization of cybersecurity professionals to work proactively to identify the cybersecurity threats and (ii) utilizing cybersecurity professionals to work with national-level CERT experts to resolve cybersecurity incidents. The first cybersecurity action is related to the ISO/IEC 27002:2022 “Threat Intelligence” (Control #5.7). In an organization with limited human resources, cyberplace managers may not have the time or opportunity to work proactively to identify the cybersecurity threats. Instead, they may choose to prioritize their time by focusing on preventing or responding to cybersecurity threats. The second cybersecurity action is related to ISO/IEC 27002:2022 “Contact with Special Interest Groups” (Control #5.6). Due to privacy reasons, organizations may be reluctant to work with experts from other cybersecurity organizations on cybersecurity incident matters, even when they lack specialist knowledge in the area of cyber resolution. By contrast, organizations with limited resources may consider tapping the skills of external expert organizations such as CERTs.

Based on the concepts and theories of place management, we propose the following hypotheses:

#### Hypothesis 1

Organizations that utilize cybersecurity professionals to work proactively to identify the cybersecurity threats are perceived as more effective in controlling cybercrimes than those organizations that do not.

#### Hypothesis 2

Organizations that utilize cybersecurity professionals to work closely with national-level CERT experts are perceived as more effective in controlling cybercrimes than organizations that do not.

### Sample and participants

We gathered a convenience sample of cybersecurity professionals working in Singapore and Australia to test our hypotheses. We included Singaporean and Australian cybersecurity professionals because we only had access to the members of the cybersecurity associations and CERT organizations from these two countries. The cybersecurity professionals were invited to participate in a vignette experimental survey study hosted in an online Qualtrics platform. We included four vignette scenarios in the survey (see Appendix for detailed descriptions). Respondents were randomly allocated only one of the four vignette scenarios by the platform. The survey instrument included questions on (i) the background of the respondent’s organization; (ii) the respondent’s perception of their organization’s effectiveness and experience in preventing cybersecurity incidents, such as ransomware; (iii) vignette scenarios and related questions; (iv) perceptions on the effectiveness of CERT experts; and (v) the respondent’s background. We included a unique identifier to ensure respondents could only participate in the survey once.

Emails containing a link to the survey were sent to members of cybersecurity associations, such as the Australian Information Security Association (AISA), ISACA Singapore Chapter, executive committee members of the Association of the Information Security Professional (AiSP), and alumni of the Singapore Institute of Technology (SIT) information security degree program. In addition, attendees of the AusCERT Cyber Security Conference 2021 were also invited to participate in the survey. Initially, there were very few responses from Australian cybersecurity professionals. Hence, in order to encourage more responses, we offered an incentive to the attendees of the AusCERT Cybersecurity Conference at our physical display booth. The attendees who participated in the survey were eligible for a lucky draw of ten AUD 50 shopping vouchers. The survey was also posted in the LinkedIn social media platform. The survey was open from May to June 2021. The emails reached an estimated 2100 potential respondents and 225 responded, with an estimated response rate of 10.1%. Our low response rate is consistent with response rates to online surveys in the literature (Van Mol 2017) and was likely because respondents were wary of phishing emails, disinterested in survey participation, or suffering from survey fatigue (Muñoz-Leiva et al. 2010; Van Mol 2017; Tourangeau et al. 2013). The low response rate could result in response bias affecting the reliability of this study (Van Mol 2017; Fan and Yan 2010). There were 213 valid responses to the survey after removing the incomplete and duplicated entries.

Table 1 contains the characteristics of the 213 respondents. Of the total respondents, 25.8% were from Australia and 67.65% from Singapore, 1.4% were from other countries while 5.2% did not report the country in which they were working. With regard to experience working in the cybersecurity industry, 49.3% of the respondents had less than six years of experience while 45.5% had six or more years and 5.2% did not report the number of years of working experience. For education, 70.9% had at least a degree in Information Technology (IT), cybersecurity, or computer science.

**Table 1:** Respondents’ characteristics

Characteristics	Frequency	
	%	(*n*)
*Country*		
Australia	25.8	55
Singapore	67.6	144
Others	1.4	3
Not reported	5.2	11
*Years of working experience*		
Less than 6 years	49.3	105
6 to 10 years	17.8	38
11 to 15 years	10.8	23
More than 15 years	16.9	36
Not reported	5.2	11
*Formal qualifications related to IT, cybersecurity, or computer science*		
Diploma	28.2	60
Degree	70.9	151
Masters	14.1	30
PhD	6.1	13
None or not reported	9.9	21
*Cybersecurity industry/professional certifications*		
One only	23.5	50
More than one	41.8	89
Not reported	34.7	74
*Cybersecurity industry/professional memberships*		
One only	31.5	67
More than one	22.1	47
Not reported	46.5	99

### Procedures

The vignettes presented respondents with a hypothetical scenario where an organization utilized a cybersecurity professional as the assigned cyberplace manager in two different ways to protect the organization’s assets and reduce opportunities for cybercrimes. We varied the vignette scenarios (see Appendix) so that the cyberplace manager in the organization was either utilized (or did not) to work proactively to identify the malicious cybersecurity threats and also utilized to work closely (or did not) with national-level CERT experts to resolve cybersecurity incidents.

Table 2 shows the four vignette scenarios that are permutations of the two cybersecurity activities performed by the cyberplace managers. In Scenario #1 (S1), the cyberplace manager is utilized to work proactively to identify the cybersecurity threats and worked closely with national-level CERT experts to resolve cybersecurity incidents. In Scenario #2 (S2), the cyberplace manager is utilized to work proactively to identify the cybersecurity threats but did not work closely with national-level CERT experts. In Scenario #3 (S3), the cyberplace manager is not utilized to work proactively to identify the cybersecurity threats but worked closely with national-level CERT experts. In Scenario #4 (S4), the cyberplace manager is not utilized to work proactively to identify the cybersecurity threats and also did not work closely with national-level CERT experts. Respondents were randomly presented one of the four vignette scenarios. See Appendix for the details of the four vignette scenarios.

**Table 2:** Vignette scenarios

	Utilized to work with national-level CERT experts to resolve cybersecurity incidents	Is not utilized to work with national-level CERT experts to resolve cybersecurity incidents
Utilized to work proactively to identify the cybersecurity threats	Scenario #1	Scenario #2
Is not utilized to work proactively to identify the cybersecurity threats	Scenario #3	Scenario #4

### Measures

After reading the assigned vignette scenarios, the respondents were asked to rate the effectiveness of the organization in achieving the cybersecurity functions: (i) *identifying* cybersecurity threats, (ii) *preventing* cybersecurity attacks, (iii) *responding* to cybersecurity incidents, (iv) *minimizing* losses from cybersecurity incidents, and (v) *recovering* from cybersecurity incidents. The responses to these vignette questions were measured in a five-point Likert scale from (i) Highly ineffective (coded 1) to (ii) Somewhat ineffective (coded 2) to (iii) Neutral (coded 3) to (iv) Somewhat effective (coded 4) to (v) Highly effective (coded 5).

### Data analysis

We ran a 2 × 2 × 5 mixed methods analysis of variance (ANOVA) to test the hypotheses. The first independent variable (IV) was a between-groups variable: working proactively to identify the cybersecurity threats (yes and no). The second independent variable (IV) was a between-groups variable: working with CERT experts to manage cybersecurity incidents (yes and no). The third IV was a within-groups variable: the type of cybersecurity function (identify, prevent, respond, minimize, and recover). The dependent variable (DV) was the perception of effectiveness of the organization in controlling cybercrimes.

## Results and analysis

We conducted all analyses used in this results section using STATA SE 16. We used a 2 (work Proactively) × 2 (work with CERT) × 5 (cybersecurity function) mixed methods ANOVA, with working Proactively and with CERT as the between-group variables and the cybersecurity functions as the within-group variable. The main effects and interactions of the variables are shown in Table 3. Skewness and kurtosis values across all vignette scenarios and cybersecurity functions were found to be within ± 0.1 and ± 2.1, respectively.

**Table 3:** Analysis of variance between work proactively, work with CERT, and cybersecurity function (*N* = 213)

Variables	df	*F*	*p*	*np*^2a^
Model	19, 1045	37.34	< 0.001	0.40
Work proactively (P)	1, 1045	609.71*	< 0.001	0.37
Work with CERT (C)	1, 1045	83.55*	< 0.001	0.07
Cybersecurity Function (F)	4, 1045	1.01	0.399	< 0.01
P x C	1, 1045	2.36	0.125	< 0.01
P x F	4, 1045	2.46*	0.044	0.01
C x F	4, 1045	0.58	0.677	< 0.01
P x C x F	4, 1045	0.71	0.584	< 0.01

*p < 0.05

^a^Partial ETA squared

**Fig. 1:**
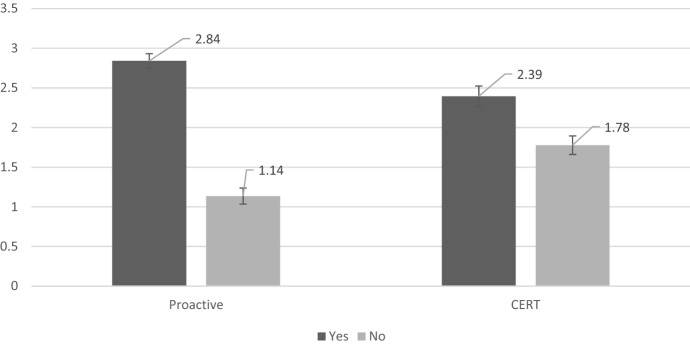
Mean for effectiveness of controlling cybercrimes for working proactively and working with CERT (+ SE bars)

We ran a three-way mixed ANOVA on the sample of 213 respondents to examine the effect of cyberplace managers working proactively to identify the threats, working with CERT, and the type of cybersecurity functions on the perception of organizations in controlling cybercrimes. There was a significant main effect in the effects of working proactively on the perception of effectiveness by organizations in controlling cybercrimes, *F*(1, 1045) = 609.71, *p* < 0.001, *np*^2^ = 0.37. Respondents perceived that organizations that utilize cyberplace managers to work proactively to identify the cybersecurity threats were more effective in controlling cybercrimes than those organizations that did not. There was a significant main effect in the effects of working with CERT on the perception of effectiveness by organizations in controlling cybercrimes, *F*(1, 1045) = 83.55, *p* < 0.001, *np*^2^ = 0.07. Respondents perceived that organizations that utilize cyberplace managers to work with CERT experts to manage cybersecurity incidents were more effective in controlling cybercrimes than those organizations that did not. Figure 1 shows the mean effectiveness of controlling cybercrimes for working proactively and working with CERT. The mean effectiveness of working proactively is 2.84 compared to the mean effectiveness of 1.14 for not working proactively. Similarly, the mean effectiveness of working with CERT is 2.39 compared to the mean effectiveness of 1.78 for not working with CERT.

**Fig. 2:**
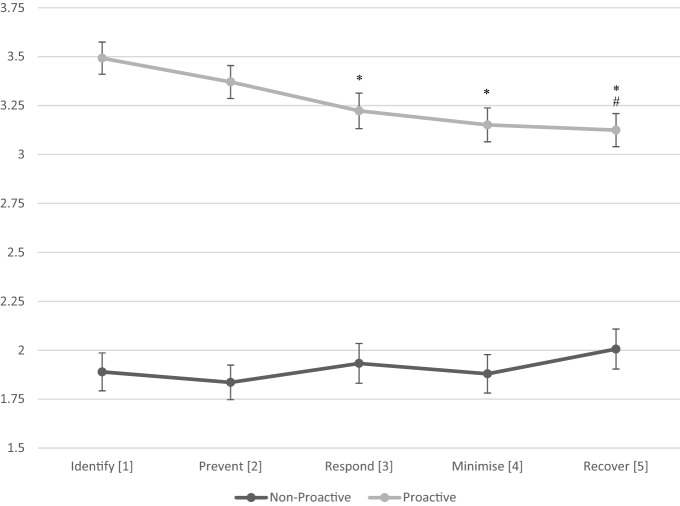
Mean plot on the effectiveness of controlling cybercrimes for working Proactively and the cybersecurity functions (Identify [1], Prevent [2], Respond [3], Minimize [4], and Recover [5]) (+ SE bars). *p < 0.05 for function Identify [1] against Respond [3], Minimize [4], and Recover [5]. ^#^*p* < 0.05 for function Prevent [2] against Recover [5]

Table 3 also shows that there was a significant interaction between the effects of working proactively and the cybersecurity functions on the perception of effectiveness by organizations in controlling cybercrimes, *F*(4, 1045) = 2.46, *p* = 0.044, *np*^2^ = 0.01. We further explored the two-way interactions of both variables using pairwise comparisons; see Table 4 below for the results. As Table 4 shows, when cyberplace managers worked proactively, there was a statistically significant difference between the function of identify [1] compared to respond [3], *p* = 0.025, minimize [4], *p* = 0.005, and recover [5], *p* = 0.002. There was no significant difference between the function of identify [1] compared to prevent [2], *p* = 0.311. There was also a statistically significant difference between the function of prevent [2] compared to recover [5], *p* = 0.039. There were no significant differences between the function of prevent [2] compared to identify [1], respond [3], minimize [4], and recover [5]. The mean scores for the proactive and non-proactive groups across the five cybersecurity functions are also presented in Fig. 2. There was a statistically significant difference in the mean scores of the identify [1] function when compared to respond [3], minimize [4] and recover [5] cybersecurity functions for the proactive groups. The standard error bars for the identify [1] function did not overlap for these three functions, indicating a significantly different distribution of the mean. There were no statistically significant differences in the mean scores across all five cybersecurity functions for the non-proactive group.

**Table 4:** Pairwise comparisons on the effects of working proactively on the cybersecurity functions (Identify [1], Prevent [2], Respond [3], Minimize [4], and Recover [5])

	Contrast	*t*	*p*
Prevent [2] vs Identify [1]	− 0.12	− 1.01	0.311
Respond [3] vs Identify [1]	− 0.27*	− 2.24	0.025
Minimize [4] vs Identify [1]	− 0.34*	− 2.85	0.004
Recover [5] vs Identify [1]	− 0.37*	− 3.08	0.002
Respond [3] vs Prevent [2]	− 0.15	− 1.23	0.22
Minimize [4] vs Prevent [2]	− 0.22	− 1.83	0.067
Recover [5] vs Prevent [2]	− 0.25*	− 2.06	0.039
Minimize [4] vs Respond [3]	− 0.07	− 0.61	0.545
Recover [5] vs Respond [3]	− 0.10	− 0.83	0.405
Recover [5] vs Minimize [4]	− 0.03	− 0.23	0.82

*p < 0.05

## Discussion

Using a vignette experimental study, this paper explored the perceived effectiveness of cyberplace managers who were proactively utilized to prevent cybercrimes compared to those organizations who failed to create the time and space for cyber professionals to prevent and control cybercrime attacks. We also examined perceptions of the effectiveness of cyberplace managers working with CERT professionals (or not). We presented four scenarios that offered permutations of an organization using and assigning cyberplace managers to prevent cybercrimes. We varied the scenarios based on whether organizations utilizing cyberplace managers to work proactively to identify the malicious cybersecurity threats and whether they worked in partnership with national-level CERT experts to manage and respond to cybersecurity incidents. The actions taken by assigned cyberplace managers included real-world examples of the types of cybersecurity work that cybersecurity professionals undertake depending on their job roles and types. For example, monitoring cybersecurity bulletins and patch notifications are typical tasks of the *Identify* cybersecurity function for “Threat Intelligence” in ISO/IEC 27002:2022.

We tested two hypotheses. H1 explored whether organizations that utilize cybersecurity professionals to work proactively to identify the cybersecurity threats are perceived as more effective in controlling cybercrimes than organizations that do not. H2 explored whether organizations that utilize cybersecurity professionals to work closely with a national-level cyber-emergency response team are perceived as more effective in controlling cybercrimes than organizations that do not. Our findings indicate that there is statistically significant support for both hypotheses. Contemporary cybersecurity professionals view organizations that have utilize assigned cyberplace managers to work specifically on cybersecurity activities (work proactively and work with CERT experts) as more effective in controlling cybercrimes than organizations that do not. Our findings also indicate that when cyberplace managers work proactively, they are perceived to be more effective in identifying cybersecurity threats compared to their ability to effectively respond, minimize losses, or recover from cybersecurity incidents. Therefore, given finite financial and personnel resources, organizations should consider utilizing their cyberplace managers to focus on these two areas of work, especially given the huge number of different types of cyber controls available.

### Comparing and contrasting physical space and cyberspace

Studies over a forty-year period since 1979 have explored the concept of place and its meaning in the physical world (Sherman et al. 1989; Madensen and Eck 2012; Weisburd 2015; Eck and Weisburd 2015). Sherman et al.’s (1989) define geographic places as fixed physical environments that can be seen completely and simultaneously using one’s naked eye. Many other researchers (Madensen and Eck 2012; for example, Eck and Weisburd 2015) have since clarified the nature and definition of places and how they are linked to crimes. However, the term “place” poses a new set of challenges when place is defined in cyberspace. Researchers such as Madensen and Eck (2012) highlight that virtual places are as real as physical spaces and that much of what we know about crime prevention at physical places can be applied to virtual places, with appropriate modifications. Others such as Reyns (2010) state that the internet, websites, chat rooms, and social networking sites are examples of places in the online environment that pose different crime prevention challenges. Ho et al.’s (2022) review indicates that the extant literature offers little explanation or elaboration on the definition of cyberplace, the differences between physical places versus cyberplaces and what place management and crime prevention tactics might block crime opportunities in cyberplaces.

Cyberspace is constructed based on data, which in its rawest form comprises bits of zeroes and ones. With the aid of the correct software and relevant knowledge, it is relatively easy to create, destroy, copy, and transform a cyberplace, such as a website, in a virtual world. The underlying world of cyberspace is also very dependent on the complex hardware that supports it. In cyberspace, the concept of space and time is, therefore, different from physical space (Miró Llinares and Johnson 2018; Yar and Steinmetz 2019; Ho et al. 2022). Ho et al. (2022) explain that in cyberspace, an IP address can be continents apart but still be accessible within milliseconds. Therefore, in cyberspace, it is easy to achieve almost real-time communication despite vast geographic distances.

Physical and online places are similar in that they both involve a range of different types of environments where people congregate and human activities take place (Eck and Clarke 2003). Street-level drug dealing in the physical world, for example, is highly dependent on a range of physical characteristics. Barnum et al. (2017) use risk terrain models to show that physical locations with fixed characteristics such as “…broken street lighting, affordable housing, foreclosures, and problem landlords were at higher risk for cannabis, heroin, and crack dealing” (2017, p. 1741). Similarly, cyber drug dealing can also be dependent on the specific characteristics of how cyberspace is constructed. Drug dealing can only happen in a virtual world if the cyberspace facilitates it, such as an interactive forum or an online gaming world that allows for both buyers or sellers to communicate. It cannot happen in a static website where users cannot communicate with one another.

However, due to the nature of cyberspace, we note some important differences between the place management of cyber and physical places. First, cyberspace is digitally constructed, and it often does not look or function like the real physical world and can be modified relatively easily compared to the physical world. The physical world is constructed in ways that see criminals seeking out amenable places rather than altering physical places to suit their needs. From a crime prevention perspective, it can take significant effort and time to change the physical world to prevent crimes from occurring. As such, drug dealers, burglars, and other types of criminals committing crimes in physical places gravitate to amenable places rather than altering place characteristics to suit their needs. In contrast, in cyberspace, a cyberplace such as a website or virtual gaming world is digitally constructed and can be modified relatively easily by criminals to make such places amenable to crime activity. It is relatively common for web or software developers to rely on popular software packages and libraries such as Java and OpenSSL to build a cyberplace such as a website. When a vulnerability is discovered in these software packages and libraries, all the cyberplaces that were constructed using them will be vulnerable to the same cyber-attacks and can be modified by cybercriminals at will. For example, the Apache log4j vulnerability was disclosed in 2021. Log4j is a popular Java library used in many software for logging purposes. The Common Vulnerability Scoring System (CVSS) rated it the highest possible threat score because of the sheer number of systems that are vulnerable and the ease with which a cyber-attacker can compromise the network (Wetter and Ringland 2021; Lim 2022). Furthermore, when a cybercriminal gains unauthorized access to the ultimate superuser or administrator account in these cyberplaces, they can re-construct the cyberplace easily using methods such as defacing the website, capturing passwords using keystroke loggers and installing a backdoor. In contrast, it is relatively more difficult to change physical places: it can take a lot of effort, time, and equipment to alter the physical environment. Criminals in the physical world, therefore, seek out existing and amenable places rather than altering the physical environment. However, in a cyberplace, with the right skillset and cybersecurity vulnerability or misconfiguration, a cybercriminal or malware can gain access to or modify a cyberplace easily in seconds with little noise or alert. In addition, the need for cyberspace infrastructures to support cyberspace results in these very infrastructures being new potential targets of crimes, leading to cyber-focused crimes such as hacking and Distributed Denial of Service (DDoS) that target these infrastructures (Ho et al. 2022). Furthermore, the entry barrier to manipulating cyberspace is relatively lower compared to the physical world. A semi-skilled cybercriminal with the right technical know-how, such as a script-kiddy, can easily download a cybertool to exploit a flawed cyberplace, like a website, for their own criminal purposes without the knowledge of the owner of the cyberplace.

Second, cyberspace’s space–time attributes enable criminals to gather and disperse quickly at a cyberplace. It also allows large numbers of criminals to congregate at many cyberplaces at once while affecting many victims at different cyberplaces at the same time. This is unlike the physical world where a criminal or victim can only be at a single place at single point of time. A physical place such as a commercial building may typically only have hundreds or thousands of visitors in a day due to geographical and distance limitations. But a website or an online game do not have such limitations and can easily host millions of online visitors in a day. All these factors further complicate the law of crime concentration (Weisburd 2015). Cyberspace’s space–time attributes also allow criminals to be less constrained by logistical costs due to geography. It is costly for a criminal to travel from Brisbane to Singapore to commit a crime but in cyberspace, there is little cost. This means that a cybercriminal can potentially cause greater impact through cyberspace. For example, a shooter can kill dozens of people in seconds but the shooter’s reach is affected and limited by the nature of the physical world (such as limited ammunition, building doors, and walls), distance, and time. In contrast, a cybercriminal is not burdened by these issues. A cybercriminal can cause ransomware attacks easily at any cyberplace anywhere simultaneously. In 2021 and 2022, for example, cybercriminals hacked and caused ransomware attacks at multiple US hospitals, leading to fears that people would die if the ransom was not paid (Paul 2022). Compared to the physical world, the impact of crimes is amplified in the online world in terms of reach and speed. Additionally, the type of cyberspace and the natural anonymity afforded to it through the use of IP addresses means that it is much harder to identify the crime and the criminal at a cyberplace than in the physical world, which in turn may decrease the effectiveness of capable guardianships (Ho et al. 2022). These marked differences between the physical world and cyberspace mean that the utilization of place managers requires organizations to create the necessary time and space for professionals to use their cyber expertise.

The two abovementioned characteristics and the nature of cyberspace can impact our understanding of the existing theories of place management. Cyberspace is vulnerable to manipulation by skilled cybercriminals. Cybercriminals can also hide easily, which can greatly amplify their reach. Therefore, we argue that most organizations need a skilled cyberplace manager—such as a cybersecurity professional—to counter this threat. Place managers with *personal*, *diffuse,* and *general* responsibilities (Felson 1995), who are unlikely to have the skillsets to monitor cyberplace effectively, are generally not suitable to perform the role of cyberplace managers.

In a bar, the bar owner, bouncer or guard, waiter, and customer could be viewed as place managers with *personal*, *assigned*, *diffuse,* and *general* responsibilities when it comes to the bar’s physical security. All of these people in a bar can play a role in preventing any threats to physical security or an altercation, although the bouncer or guard (assigned), who is likely to be more skilled and trained, is best equipped for the role. However, this may not be possible in cyberplace. A person can perceive a cyberplace such as a website or an online forum only through its deliberately designed interface. However, there are other parts of the cyberplace that are not so easily perceived, such as the underlying operating systems and networks that support the cyberplace. On a website, the typical web owner (*personal*), web administrator (*diffuse*), and online user (*general*) may detect simple cybersecurity attacks when the attacks are visually perceptible, such as a website defacement by script kiddies. But they are unlikely to detect a cyberattack that is not visually perceptible such the Apache log4j vulnerability or a web Injection attack or a Broken Access Control attack (OWASP n.d.). They are also unlikely to have the skillsets and training to detect sophisticated cyber-attacks such as an Advanced Persistent Threats attack campaign or ransomware attacks from nation state sponsors or professional cybercriminal syndicates. We argue, therefore, that the effectiveness of a place manager with *personal*, *diffuse,* and *general* responsibilities in a cyberspace is limited due to the nature of cyberspace. We propose that, as the use of online services grows, especially due to the COVID-19 pandemic (UNCTAD 2021), we need *assigned* place managers who are specially tasked and trained with cyberplace management responsibilities. The growing employment of cybersecurity professionals as the assigned cyberplace managers (US Bureau of Labor Statistics n.d.) points to this reality.

We also argue that the *proprietary*, *proximal,* and *pooled* descriptions of places (see Madensen and Eck 2012) may need updating in relation to cyberplace. Madensen and Eck (2012) explain that *proximal* places are small groups of *proprietary* places that are housed relatively close in space, whether in the physical world or in the virtual world, and that “spatial immediacy may allow one proprietary place to impact crime at another within a specific proximal place.” (2012, p. 5). *Pooled* places are higher levels of *proprietary* place aggregation such as a neighborhood (Madensen and Eck 2012). These terms may make sense for physical places. Buildings next to each another in the same neighborhood in Brisbane (proximal) have “spatial immediacy” while the neighborhood in Brisbane is far apart enough from another neighborhood in Singapore (*pooled*) that it can pose logistical and geographical challenges for a criminal and hence there is no “spatial immediacy.” But in cyberspace, due to its space–time attributes, there may not be any significant difference between the terms *proximal* and *pooled* because “spatial immediacy” applies everywhere in cyberspace. There is not much difference in logistical and geographical reach for a criminal in cyberspace. Therefore, in terms of reach, there is the same “spatial immediacy” in any cyberplace regardless of whether a set of webpages (*proximal*) within website "Website-A" is viewed as closer to one another compared to webpages located in another website "Website-B" (*pooled)*. This is especially true when these websites "Website-A" and "Website-2" are created using the same underlying software packages or libraries because they then share the same set of vulnerabilities (such as Apache log4j) so that the ease and time to exploit them is the same regardless of their virtual location (*proximal* or *pooled*).

## Limitations

Several limitations are relevant to our study. First, our vignette experimental study uses a non-probability and convenience sample from only Australia and Singapore. Second, our sample size of 213 cybersecurity professionals is relatively small. Third, our response rate is also relatively low at 10.1%, although this is the prevailing response rate for online surveys (Van Mol 2017). All these factors may affect the external validity and generalizability of our study. We recommend that future studies examine these domains with additional samples. Nevertheless, our sample is likely to be somewhat representative of the demographics of typical cybersecurity professionals: the (ISC)2 Cybersecurity Workforce Study reported that 67% of their respondents had degrees in IT, computer science, or cybersecurity ((ISC)2 2021). Similarly, 71% of our survey respondents had a degree in IT, computer science, or cybersecurity. The (ISC)2 report also found that 50% of their respondents were currently pursuing or planning to pursue cybersecurity industry certifications; 42% of the respondents in our survey reported having at least one such certification. Fifth, our study cannot establish causal relationships due to the cross-sectional nature of the study. Finally, we only explored two of the 93 cybercrime prevention control tactics that are available in ISO/IEC 27002:2022. However, this was planned because of the anticipated small sample size.

## Concluding remarks

Our research findings indicate statistically significant support that when organizations utilize cybersecurity professionals to work proactively or work closely with CERT experts, they are perceived as more effective in controlling cybercrimes than those organizations that do not. Therefore, organizations may find great value in utilizing their assigned cyberplace managers on these two cybercrime prevention control tactics. Our vignette experimental study investigated only two of the many cybercrime prevention control tactics that could be implemented by assigned cyberplace managers: working proactively to identify the cybersecurity threats and working with a national-level CERT to manage cybersecurity incidents. These two cybercrime prevention control tactics form a small subset of the 93 control tactics available in ISO/IEC 27002:2022. There are also other control tactics from other popular cybersecurity industry standards or advisories. Some of these cybercrime prevention control tactics can be complicated to implement and require a tremendous amount of time and resources. Proactively monitoring cybersecurity threats can require a Security Operation Center (SOC) comprising of several cybersecurity professionals. Therefore, there is ample room to explore the effectiveness of other types of work and cybercrime prevention control tactics that assigned cyberplace managers could undertake.

We concur with past research arguing that place management is a promising, although underutilized, SCP technique (Douglas and Welsh 2022). We acknowledge research calls to provide evaluative evidence of the effectiveness of SCP and place managers in preventing crimes and cybercrimes (Welsh et al. 2010; Maimon and Louderback 2019; Douglas and Welsh 2020; Ho et al. 2022). We recognize that the definition of place using the traditional ideas of physical space and place managers is well defined (Sherman et al. 1989; Felson 1995; Madensen and Eck 2012; Weisburd 2015; Eck and Weisburd 2015) and welcome recent attempts to extend place management from its traditional physical space to cyberspace (Reyns 2010; Madensen and Eck 2012; Maimon et al. 2022). However, we find that there is room to explore the differences between physical space and cyberspace and the impact of these differences on existing place management theories. Understanding and taking into account the inherent characteristics of cyberspace can lead to an improvement in how place management and place managers can prevent cybercrimes more effectively.

## Appendix: List of vignette scenarios

### Vignette Scenario #1 (Proactive + Work with CERT)

Firelight Ltd recently hired Alex as a member of the cybersecurity team to discourage cybercrimes, reduce opportunities for cybercriminal events and secure the company’s cybersecurity assets. The team focuses on preventing, detecting, resolving, and managing cybersecurity matters. Examples include developing and disseminating cybersecurity policies, developing and maintaining awareness of cybersecurity threats, and working with other departments and users to prevent, detect, resolve, and manage cybersecurity threats.

Alex’s primary role in the team is to proactively identify the malicious cybersecurity threats. For example, Alex regularly monitors cybersecurity bulletins, patch notifications, potential phishing emails from cybersecurity threat intelligence services, CERTs, and so on. Upon discovery of malicious cybersecurity threats, Alex would proactively take action to block malicious cybersecurity threats, such as configuring firewalls, mail filters and IDS/IPS, to block suspicious IPs and URLs.

Alex spends a few hours a week, working closely with national-level CERTs, such as AusCERT. For example, Alex would work with them to resolve cybersecurity incidents, such as ransomware attacks in the company.

### Vignette Scenario #2 (Proactive + Do NOT Work with CERT)

Firelight Ltd recently hired Alex as a member of the cybersecurity team to discourage cybercrimes, reduce opportunities for cybercriminal events and secure the company’s cybersecurity assets. The team focuses on preventing, detecting, resolving, and managing cybersecurity matters. Examples include developing and disseminating cybersecurity policies, developing and maintaining awareness of cybersecurity threats, and working with other departments and users to prevent, detect, resolve, and manage cybersecurity threats.

Alex’s primary role in the team is to proactively identify the malicious cybersecurity threats. For example, Alex regularly monitors cybersecurity bulletins, patch notifications, potential phishing emails from cybersecurity threat intelligence services, CERTs, and so on. Upon discovery of malicious cybersecurity threats, Alex would proactively take action to block malicious cybersecurity threats, such as configuring firewalls, mail filters, and IDS/IPS, to block suspicious IPs and URLs.

For privacy reasons, the company does not like external parties to know of the type of cybersecurity threats that they face or even the actual cybersecurity incidents that do successfully occur. As such, the company does not work with any national-level CERTs, such as AusCERT. If there are any cybersecurity incidents, such as ransomware attacks in the company, the company will have to resolve it on their own.

### Vignette Scenario #3 (NOT Proactive + Work with CERT)

Firelight Ltd recently hired Alex as the only cybersecurity professional in the company to discourage cybercrimes, reduce opportunities for cybercriminal events, and secure the company’s cybersecurity assets. As the only cybersecurity professional, Alex has a lot of work to do, ranging from putting in place cybersecurity policies to educating the company’s users and departments about safe cybersecurity practices.

Due to the heavy workload, Alex could not spare a lot of time to proactively identify or block malicious cybersecurity threats. For example, Alex does not regularly monitor cybersecurity bulletins or configure firewalls, mail filters, and IDS/IPS, to block suspicious IPs and URLs.

Alex spends a few hours a week, working closely with national-level CERTs, such as AusCERT. For example, Alex would work with them to resolve cybersecurity incidents, such as ransomware attacks in the company.

### Vignette Scenario #4 (NOT Proactive + Do NOT Work with CERT)

Firelight Ltd recently hired Alex as the only cybersecurity professional in the company to discourage cybercrimes, reduce opportunities for cybercriminal events, and secure the company’s cybersecurity assets. As the only cybersecurity professional, Alex has a lot of work to do, ranging from putting in place cybersecurity policies to educating the company’s users and departments about safe cybersecurity practices.

Due to the heavy workload, Alex could not spare a lot of time to proactively identify or block malicious cybersecurity threats. For example, Alex does not regularly monitor cybersecurity bulletins or configure firewalls, mail filters, and IDS/IPS, to block suspicious IPs and URLs.

For privacy reasons, the company does not like external parties to know of the type of cybersecurity threats that they face or even the actual cybersecurity incidents that do successfully occur. As such, the company does not work with any national-level CERTs, such as AusCERT. If there are any cybersecurity incidents, such as ransomware attacks in the company, the company will have to resolve it on their own.
